# Expanding Role of Dopaminergic Inhibition in Hypercapnic Responses of Cultured Rat Carotid Body Cells: Involvement of Type II Glial Cells

**DOI:** 10.3390/ijms21155434

**Published:** 2020-07-30

**Authors:** Erin M. Leonard, Colin A. Nurse

**Affiliations:** Department of Biology, McMaster University, 1280 Main St. West, Hamilton, ON L8S 4K1, Canada; nursec@mcmaster.ca

**Keywords:** carotid body, dopamine, sulpiride, purinergic signalling, type II cells, petrosal neurons

## Abstract

Dopamine (DA) is a well-studied neurochemical in the mammalian carotid body (CB), a chemosensory organ involved in O_2_ and CO_2_/H^+^ homeostasis. DA released from receptor (type I) cells during chemostimulation is predominantly inhibitory, acting via pre- and post-synaptic dopamine D2 receptors (D2R) on type I cells and afferent (petrosal) terminals respectively. By contrast, co-released ATP is excitatory at postsynaptic P2X2/3R, though paracrine P2Y2R activation of neighboring glial-like type II cells may boost further ATP release. Here, we tested the hypothesis that DA may also inhibit type II cell function. When applied alone, DA (10 μM) had negligible effects on basal [Ca^2+^]_i_ in isolated rat type II cells. However, DA strongly inhibited [Ca^2+^]_i_ elevations (Δ[Ca^2+]^_i_) evoked by the P2Y2R agonist UTP (100 μM), an effect opposed by the D2/3R antagonist, sulpiride (1–10 μM). As expected, acute hypercapnia (10% CO_2_; pH 7.4), or high K^+^ (30 mM) caused Δ[Ca^2+^]_i_ in type I cells. However, these stimuli sometimes triggered a secondary, delayed Δ[Ca^2+^]_i_ in nearby type II cells, attributable to crosstalk involving ATP-P2Y2R interactions. Interestingly sulpiride, or DA store-depletion using reserpine, potentiated both the frequency and magnitude of the secondary Δ[Ca^2+^]_i_ in type II cells. In functional CB-petrosal neuron cocultures, sulpiride potentiated hypercapnia-induced Δ[Ca^2+^]_i_ in type I cells, type II cells, and petrosal neurons. Moreover, stimulation of type II cells with UTP could directly evoke Δ[Ca^2+^]_i_ in nearby petrosal neurons. Thus, dopaminergic inhibition of purinergic signalling in type II cells may help control the integrated sensory output of the CB during hypercapnia.

## 1. Introduction

In mammals, the main peripheral chemoreceptors are the carotid bodies (CBs), bilaterally located at the bifurcation of the common carotid artery, where they acutely monitor blood levels of O_2_, CO_2_, and pH [1,2]. In conditions of O_2_ deficiency (hypoxia) or CO_2_ excess (hypercapnia), the CBs initiate compensatory respiratory and cardiovascular reflex responses so as to regain homeostasis and protect vital organs such as the brain. The O_2_- and CO_2_/H^+^-sensitive detectors, known as glomus or type I cells, occur in clusters and share an intimate association with neighboring glial-like, sustentacular type II cells along with the abutting sensory nerve terminals [1,3]. During chemotransduction, type I cells depolarize and release excitatory and inhibitory neurotransmitters that shape the afferent output to the brainstem [1,3,4,5]. Among the excitatory neurotransmitters, ATP and adenosine are the best studied and these can act on both pre- and post-synaptic purinergic receptors [3,4,5,6,7]. On the other hand, with the exception of rabbit, dopamine (DA) is inhibitory in most species and for many years has provided a convenient assay for the secretory functions of type I cells during chemoexcitation [1,4,8,9]. Moreover, an understanding of the physiological role of DA has been greatly aided by the numerous biochemical, pharmacological, immunohistochemical, and molecular studies characterizing both pre- and post-synaptic DA receptors in the CB of several mammalian species including humans [1,2,4,8,10,11,12].

In general, dopamine D2 receptors (D2R) located on postsynaptic petrosal chemoafferent terminals and presynaptic type I cells appear to be the dominant form expressed in the CB of most species, though D1R mRNA has also been detected by RT-PCR and in situ hybridization [10,11,13]. When activated, postsynaptic D2R on petrosal terminals inhibit action potential firing [2,4,8], and a recently-proposed mechanism involves modulation of hyperpolarization-activated, cyclic nucleotide-gated HCN channels [14]. The presynaptic D2 autoreceptors on type I cells appear to inhibit intracellular Ca^2+^ signalling and neurotransmitter release via negative feedback modulation of voltage-gated Ca^2+^ channels (VGCC) [4,15,16,17]. Given the clustered arrangement of type I/type II cells, DA actions following its release during chemotransduction are likely to involve paracrine interactions with neighboring type I cells and potentially, the adjoining glial-like type II cells.

In the present study, we used rat CB cultures to investigate whether type II glial cell signalling might be influenced by the paracrine actions of DA, and perhaps contribute to the overall inhibitory pathways in the CB. The rationale evolved from previous studies demonstrating that several type I cell paracrine signals such as ATP, angiotensin II, and 5-HT enhance intracellular Ca^2+^ signalling in type II cells, potentially leading to the further release of ATP via pannexin-1 channels [18,19,20,21,22,23]. Given that ‘push-pull’ mechanisms generally seem to regulate the overall CB output [24,25], the question arose whether inhibitory neuromodulators such as DA could suppress Ca^2+^ signalling in type II cells and therefore their ability to release ATP. Interestingly, we found that DA does indeed inhibit purinergic signalling in type II cells and that this pathway may play an active role during hypercapnia. Finally, using functional cocultures of CB type I/type II cells and dissociated petrosal neurons, combined for the first time with simultaneous Ca^2+^ imaging of all three cell types, we examined the role of DA and type II cells in the integrated CB chemosensory response during hypercapnia. In addition, because there is strong evidence supporting a role for ATP release from CNS glial cells (i.e., astrocytes) in central CO_2_ chemoreception [26], we used the coculture model to test whether selective stimulation of type II cells could activate nearby petrosal neurons.

## 2. Results

In the experiments described below, type II glial cells were first tentatively identified by their characteristic elongated morphology and confirmed by the presence of a rapid and robust increase in intracellular Ca^2+^ (Δ[Ca^2+^]_i_) upon stimulation with the P2Y2R agonist, UTP [18,27]. These properties are unique to type II cells in dissociated CB cultures thereby allowing their positive identification. By contrast, chemoreceptor type I cells were identified by the presence of a rapid response to isohydric hypercapnia (10% CO_2_; pH = 7.4) and/or the depolarizing stimulus high (30 mM) K^+^. In previous studies, type II cells sometimes responded to either hypercapnia or hypoxia, however, such ‘secondary’ responses were delayed and indirect, attributable to paracrine stimulation by chemical signals (e.g., ATP) released from neighboring type I cells [22]. In the present study, hypercapnia was routinely used as the chemostimulus because, under our experimental conditions, it evoked ‘secondary’ type II cell Ca^2+^ responses that were generally larger and more frequent, and therefore more amenable to pharmacological manipulations [22,27].

### 2.1. Dopamine Attenuates UTP-Evoked Intracellular Ca^2+^ Responses in Type II Cells

**Figure 1:**
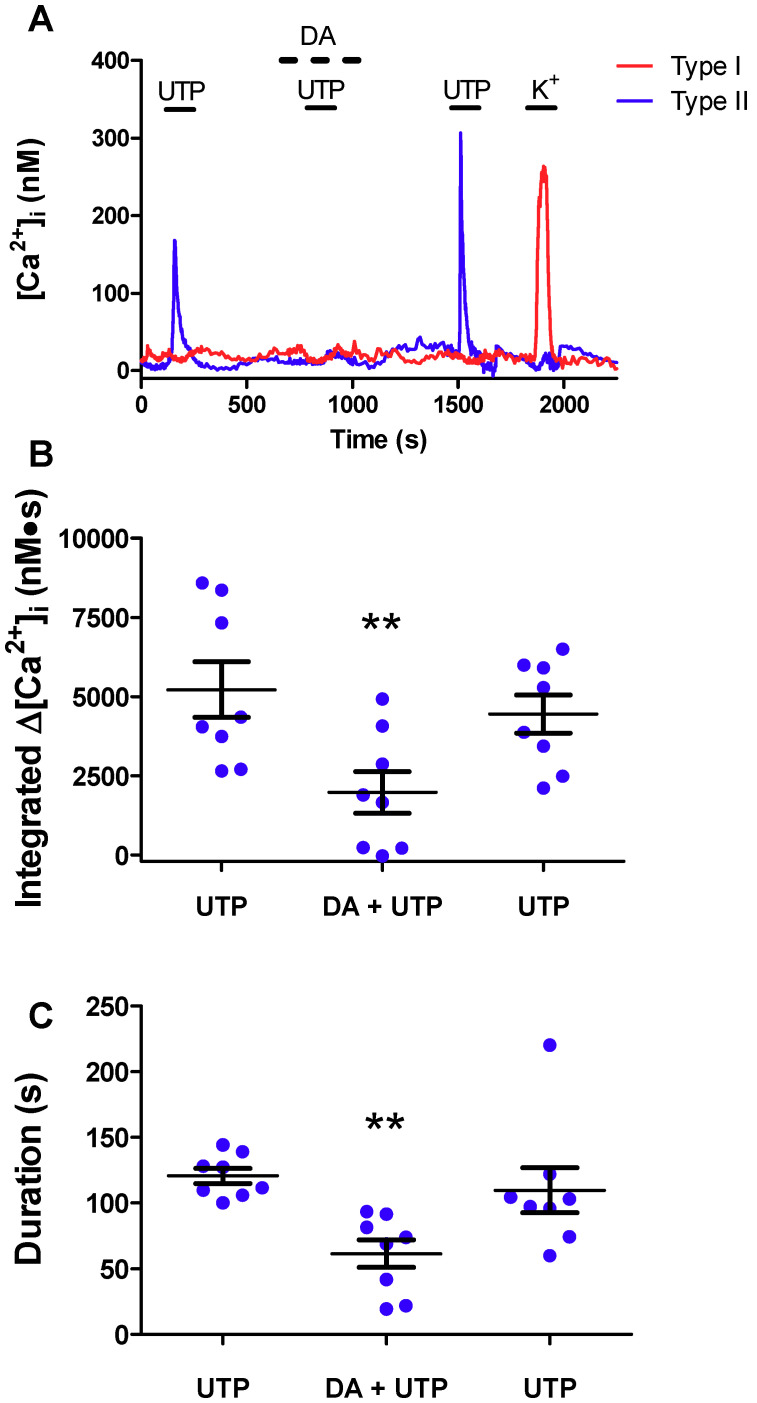
Dopamine attenuates purinergic signaling in type II cells. (**A**) Representative trace showing the reduction of the intracellular Ca^2+^ ([Ca^2+^]_i_) response to UTP (100 µM) during application of DA (10 µM) in type II cells (blue trace); contrast the type I cell (red trace) which only responded to high K^+^. (**B**) Summary data of UTP-evoked integrated [Ca^2+^]_i_ (nM∙S) response before, during, and after DA perfusion (n = 8 dishes/group, 10–25 cells sampled per dish). In (**B**) 221 of the 298 type II cells showed a reduction in the UTP response in the presence of DA. (**C**) Mean duration (s) of the UTP-evoked [Ca^2+^]_i_ response in type II cells before, during, and after DA (10 µM) perfusion. Data were analysed using a one-way repeated measures analysis of variance (ANOVA) followed by Tukey’s post hoc test; ** signifies a *p* value of < 0.01. Values are means ± S.E.M.; n = 8 dishes.

We first imaged dissociated rat CB cultures to identify cells that responded to the P2Y2R agonist UTP or high K^+^. As exemplified in Figure 1A, UTP (100 μM) evoked a rise in intracellular Ca^2+^ in a type II, but not type I, cell. By contrast, the type I, but not type II, cell showed a robust Δ[Ca^2+^]_i_ when stimulated with high K^+^ (30 mM). Though perfusion of the same culture with 10 μM dopamine (DA) alone had no effect on basal [Ca^2+^]_i_ in either cell type, the UTP-evoked Δ[Ca^2+^]_i_ in the type II cell was markedly inhibited by DA (Figure 1A). Summary data of the UTP-evoked Δ[Ca^2+^]_i_ responses integrated over time before, during, and after DA perfusion are shown in Figure 1B for several groups of type II cells (n = 8 dishes, 10–25 cells sampled per dish). In these experiments, DA significantly (*p* < 0.01) inhibited the UTP-evoked integrated Δ[Ca^2+^]_i_ (mean inhibition by ~70%) as well as the duration of the intracellular Ca^2+^ signal (Figure 1C; mean inhibition by ~50%). Of the >300 UTP-sensitive type II cells examined in this study a significant proportion (~75%) was sensitive to DA inhibition.

### 2.2. Reversal of Dopaminergic Inhibition of P2Y2R-Mediated Ca^2+^ Signalling in Type II Cells by Sulpiride, a D2/3 Receptor Antagonist

**Figure 2:**
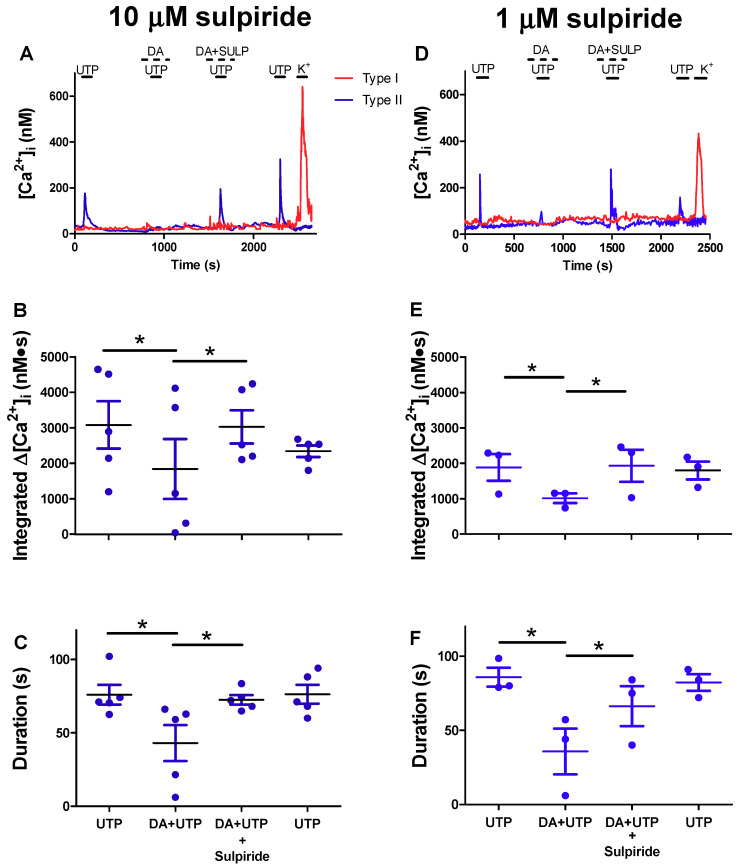
Sulpiride, a D2/3R antagonist, reverses the inhibitory effect of dopamine on the UTP-evoked intracellular Ca^2+^ rise in type II cells. (**A**,**D**) Representative type I and type II cell traces showing the [Ca^2+^]_i_ response to UTP (100 µM), UTP + DA (10 µM), UTP + DA +,Sulpiride (SULP; 10 µM (**A**), 1 µM (**D**)), and UTP alone (after washout of DA and SULP). Note Sulpiride reversed the DA inhibition of UTP-evoked [Ca^2+^]_i_ response in the type II cell; the type I cell only responded to high K^+^. Summary data of the UTP-evoked integrated [Ca^2+^]_i_ (nM∙s) (**B**,**E**) and duration of the [Ca^2+^]_i_ responses (**C**,**F**) in type II cells before, during, and after exposure to DA, or DA plus Sulpiride (n = 3–5 dishes/group, 10–15 cells sampled per dish). In these experiments, 52 of the 101 cells showed both a reduction in the UTP-evoked response in the presence of DA and subsequent recovery of the response during co-application with Sulpiride. Data were analysed using a one-way repeated measures analysis of variance (ANOVA) followed by Tukey’s post hoc test; * signifies a *p* value of < 0.05. Values are means ± S.E.M.

The inhibitory effects of DA at the CB chemosensory complex have been attributed largely to the presence of both pre- and post-synaptic D2 receptors (D2R) [8,10,15,17]. We therefore tested the effects of sulpiride, a D2R antagonist, on UTP-evoked intracellular Ca^2+^ signalling in type II cells. As exemplified in Figure 2A,D, the presence of sulpiride (both 10 and 1 μM) reversed the inhibitory effects of DA on UTP-evoked Ca^2+^ signalling in a type II cell. Summary data of the time-integrated and duration of the UTP-evoked Δ[Ca^2+^]_i_ responses in type II cells before, during, and after exposure to DA, or DA plus sulpiride, are shown in Figure 2B,E and Figure 2C,F, respectively. Note that in Figure 2B,C,E,F, the dopaminergic inhibition of P2Y2R-mediated Ca^2+^ signalling was largely suppressed or reversed in the presence of sulpiride (n = 3–5 dishes, 10–15 cells sampled per dish; *p* < 0.05). Also, when present alone, sulpiride had no effect on the basal intracellular Ca^2+^ levels in type II cells at the concentrations used, suggesting it did not cause a non-specific elevation in intracellular Ca^2+^ transients in Figure 2. These data suggest that D2-like receptors on type II cells may also contribute to the overall inhibitory effects of DA at the carotid body chemoreceptor complex.

### 2.3. Effects of Sulpiride on Paracrine Signalling between Type I Chemoreceptor Cells and Type II Glial Cells

During chemotransduction type I cells release several neurotransmitters including ATP and DA whose effects in the rat CB are predominantly excitatory and inhibitory respectively [3,4,5,7,17,28,29]. It is proposed that ATP released from type I cells may lead to its further release from neighboring type II cells following paracrine stimulation of P2Y2R and Ca^2+^-dependent activation of ATP-permeable pannexin I channels [5,20,22,29]. These studies, together with the above findings, raise the question whether DA released from type I cells during chemotransduction can inhibit the excitatory effects of co-released ATP on neighboring type II cells. To address this, CB cultures were first exposed to a hypercapnic stimulus (10% CO_2_; pH ~7.4) and intracellular Ca^2+^ transients were monitored simultaneously in type I and neighboring type II cells before, during, and after exposure to 10 μM sulpiride. This stimulus was routinely used because it often evoked large Ca^2+^ responses in type I cells and crosstalk from type I to type II cells was detected more frequently [20,22,27]. Though acid hypercapnia is the more relevant physiological stimulus, we used isohydric hypercapnia because acid hypercapnia tends to underestimate intracellular Ca^2+^ levels in type I cells due to the pH sensitivity of the K_d_ for fura-2 [27].

**Figure 3:**
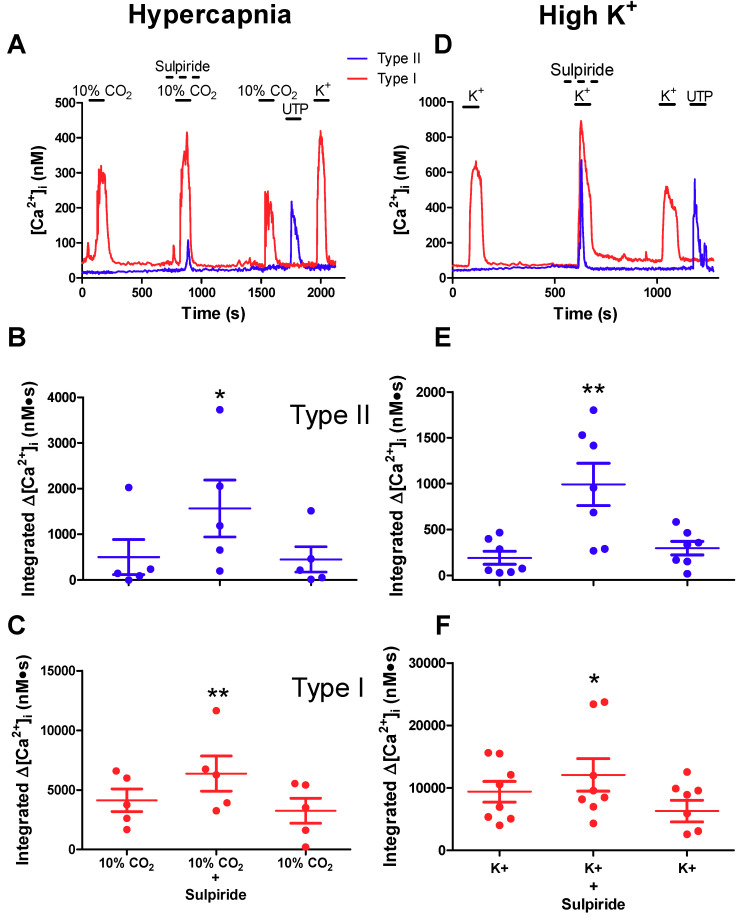
Sulpiride potentiates crosstalk among type I and type II cells during hypercapnia and high K^+^ exposure. Representative traces showing sulpiride-mediated potentiation of type I cell [Ca^2+^]_i_ responses and the unveiling or potentiation of the delayed type II cell responses during stimulation with hypercapnia (**A**) and high K^+^ (**D**). Summary data of integrated [Ca^2+^]_i_ responses (nM∙s) to hypercapnia are shown for type II (**B**) and type I (**C**) cells; n = 5 separate cultures/group, 3–25 cells sampled per dish. Similar data for the high K^+^ stimulus are shown for type II (**E**) and type I (**F**) cells; n = 7 separate cultures/group, 3–25 cells sampled per dish). Data were analysed using a one-way repeated measures analysis of variance (ANOVA) followed by Tukey’s post hoc test; *, and ** signifies a *p* value of < 0.05 and 0.01, respectively. Values are means ± S.E.M.

As exemplified in Figure 3A, exposure to hypercapnia alone caused a rapid and robust Δ[Ca^2+^]_i_ in a type I cell that was part of a cell cluster; note that the neighboring type II cell (identified by the presence of a UTP-induced Ca^2+^ transient) was unresponsive to hypercapnia. However, when the hypercapnic stimulus was re-applied to the same cells, but in the presence of sulpiride (10 μM), there was a potentiation of the type I cell Ca^2+^ response and the unveiling of a delayed Δ[Ca^2+^]_i_ in the type II cell (Figure 3A). Summary data of the rapid and delayed Δ[Ca^2+^]_i_ transients for several groups of type II and type I cells are shown in Figure 3B,C, respectively, when the hypercapnic stimulus was applied before, during, and after perfusion with sulpiride. Note the significant potentiation of the integrated Δ[Ca^2+^]_i_ response in both type I and type II cells when the hypercapnic stimulus was applied in the presence of sulpiride (n = 4–5 dishes; 3–25 cells sampled per dish; *p* < 0.05 in type II cells and *p* < 0.01 in type I cells). We attribute the potentiation of the type I cell Ca^2+^ response by sulpiride to the prevention or removal of autocrine-paracrine negative feedback inhibition mediated by released DA acting on D2R on type I cells [15,17]. On the other hand, the potentiation of the type II cell Ca^2+^ response was likely due to sulpiride-mediated disinhibition, i.e., the suppression or removal of DA inhibitory effects on ATP-P2Y2R -mediated Ca^2+^ signalling during crosstalk from type I to type II cells. It should be noted that, in separate experiments where hypercapnia was applied three times to the same cells with no addition of sulpiride, there was an average decrease of ~6% in the 2^nd^ vs. 1^st^ hypercapnic response (data not shown) and this variation was not statistically significant (*p* > 0.05; n = 4 cultures). These data further emphasize that the increased hypercapnic response in the presence of sulpiride is due to the effects of the drug.

To obtain further support for the posit that DA inhibits ATP-mediated crosstalk between type I and type II cells we used a high K^+^ stimulus to evoke a more predictable robust release of ATP and DA from type I cells [9,17,22,30]. Indeed, as exemplified in Figure 3D, high K^+^ (30 mM) caused a robust Δ[Ca^2+^]_i_ in a type I, but not type II, cell when applied alone. By contrast, when the high K^+^ stimulus was re-applied in conjunction with sulpiride to the same cells there was an unveiling of a delayed, robust Ca^2+^ response in the type II cell. This effect was reversible after washout of sulpiride (Figure 3D). Summary data showing the sulpiride-induced potentiation of the delayed and rapid Δ[Ca^2+^]_i_ transients for several groups of type II and type I cells are shown in Figure 3E,F during high K^+^ application (n = 7; *p* < 0.01 in type II cells and *p* < 0.05 in type I cells).

We also tested the effects of hypoxia (PO_2_ ~15–20 mmHg), though under our experimental conditions the intracellular Ca^2+^ responses in type I cells tended to be smaller and were detectable at a lower frequency than for high K^+^ or hypercapnic stimuli [22,27]. Nevertheless, in two cases, we observed a similar potentiation of the hypoxia-induced Δ[Ca^2+^]_i_ in both type I and neighboring type II cells in the presence of sulpiride. However, the responses were poorly reversible after washout of the drug (data not shown). Taken together, the combined data from these studies suggest that during chemoexcitation the paracrine excitatory effect of ATP-P2Y2R signalling on Ca^2+^ transients in type II cells is blunted by the concurrent paracrine action of co-released DA acting via sulpiride-sensitive DA receptors.

### 2.4. Pre-Treatment with Reserpine to Deplete Dopamine Stores Facilitates Crosstalk from Type I to Type II Cells

**Figure 4:**
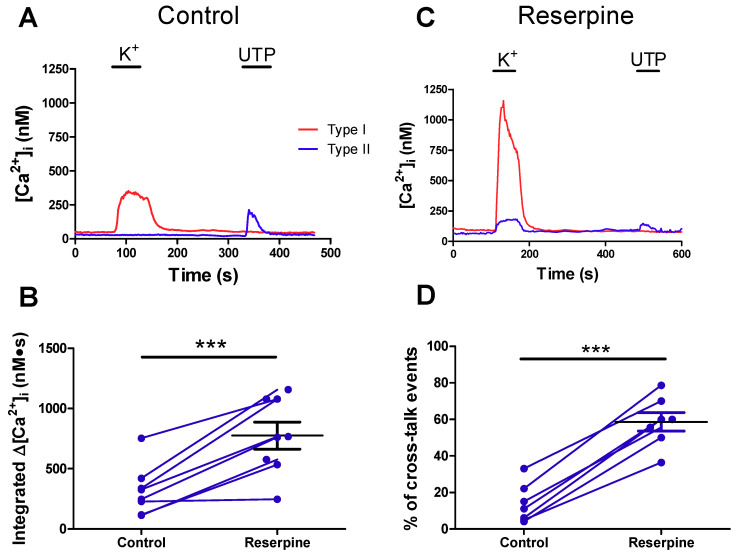
Depleting vesicular type I cell dopamine stores using reserpine enhances crosstalk from type I to type II cells. Representative traces showing intracellular Ca^2+^ responses of type I and type II cells from a control (**A**) and (**C**) reserpine-treated (**C**) ‘sister’ culture as a result of exposure to high K^+^ and UTP. (**B**) Integrated [Ca^2+^]_i_ in type II cells in response to high K^+^ depolarization of neighboring type I cells in control versus reserpine-treated sister cultures. Blue lines connect mean values from type II cells imaged on the same day from paired ‘sister’ cultures. (**D**) Percent (%) of type II cells displaying crosstalk events during from high K ^+^ depolarization of type I cells in sister cultures. In B, * denotes significant difference between control and reserpine-treated cells, evaluated using Student’s t-test (* = *p* < 0.05). In D, a one-way ANOVA followed by Tukey’s post hoc test was used; *** *p* < 0.001. Values are means ± S.E.M.; n = 8 dishes, 3–20 cells tested per dish.

The above findings led to the prediction that removal of dopaminergic inhibitory pathways by DA store depletion should facilitate crosstalk from type I to type II cells. We therefore exposed CB cultures to reserpine, an inhibitor of vesicular monoamine transporter (VMAT) that is known to deplete catecholamines from type I and other sympathoadrenal cells in vivo and in vitro [31,32]. Calcium transients were monitored in control cultures and reserpine-treated sister cultures (45 min exposure to 1 μM reserpine prior to recording) in paired groups on the same day. To optimize crosstalk, type I cells were depolarized using the high K^+^ stimulus. Representative traces of the resulting Ca^2+^ transients in type I and neighboring type II cells in a control and reserpine-treated ‘sister’ culture are shown in Figure 4A,C. As predicted, the delayed type II cell Ca^2+^ responses were larger and occurred more frequently in reserpine-treated cultures compared to control ‘sister’ cultures (Figure 4B,D). Though not studied in detail, the delayed Ca^2+^ responses seen in type II cells in reserpine-treated cultures were sensitive to suramin (100 μM; n = 2, data not shown; see also 22), suggesting that purinergic signalling contributed significantly to crosstalk in these conditions, as previously demonstrated for control cultures [22]. Type I cell responses were not significantly potentiated in reserpine-treated cultures (data not shown).

### 2.5. Role of Dopaminergic Inhibition at the Reconstituted Chemosensory Complex Consisting of Petrosal Neurons, Type I Cells, and Type II Cells In Vitro

**Figure 5:**
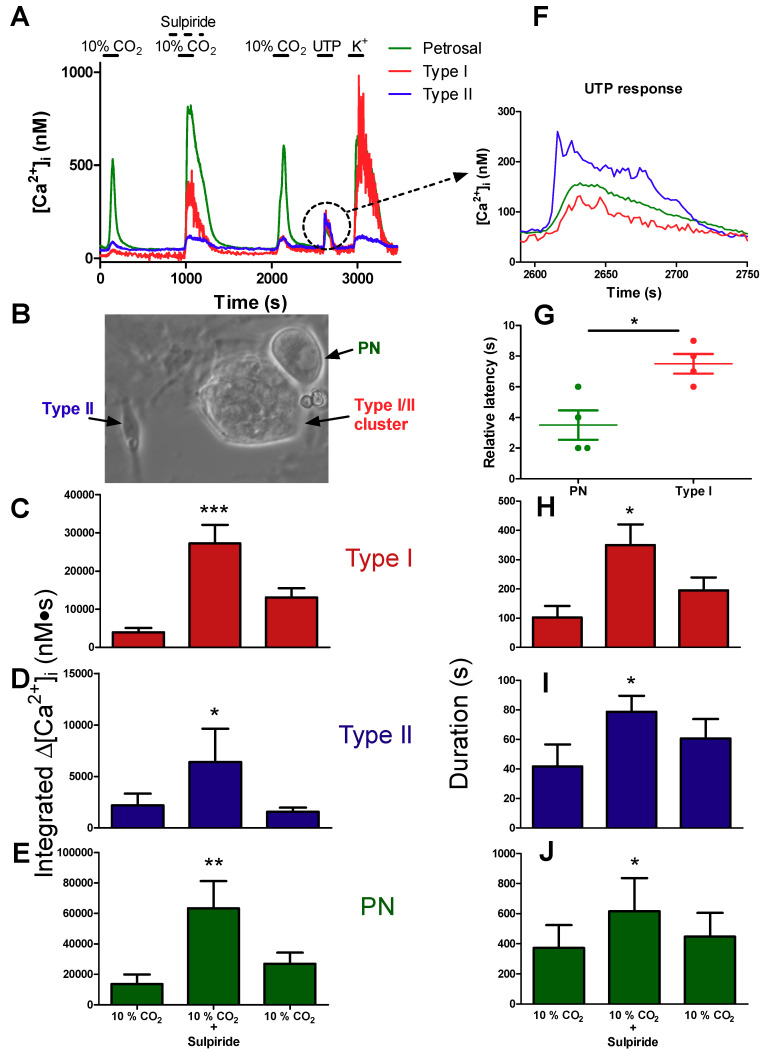
Sulpiride enhances hypercapnia-evoked responses in type I cells, type II cells, and petrosal neurons during simultaneous Ca^2+^ imaging in functional cocultures. (**A**) Representative traces showing the [Ca^2+^]_i_ responses in a type I cell (red), type II cell (blue), and petrosal neuron (green), recorded simultaneously during hypercapnia (10% CO_2_) in a carotid body coculture. (**B**) Typical phase contrast micrograph of a coculture showing a petrosal neuron (PN) adjacent to a type I/type II cell cluster; a solitary, isolated type II cell with its characteristic elongated shape is also shown (left). In A, note the marked potentiation of the hypercapnic responses in all 3 cell types when sulpiride was present, as well as the responses of the 3 cells to UTP. The UTP-evoked responses (dotted circle in **A**) are enlarged in (**F**) to reveal differences in response latency. Summary data of response latency (sec) of PN and type I cells relative to that in type II cell are shown in (**G**); note PN responses precede those in type I cells during direct stimulation of type II cells with UTP (*p < 0.05; n = 4 separate cultures). Histograms (**C**–**E,H**–**J**) show integrated [Ca^2+^]_i_ response (nM∙s) and mean duration of the [Ca^2+^]_i_ response during hypercapnia in type I cells (**C**,**H**), type II cells (**D**,**I**), and PN (**E**,**J**); n = 3 separate cultures/group. Data were analysed using a one-way repeated measures analysis of variance (ANOVA) followed by Tukey’s post hoc test; (*, **, and *** signifies a *p* value of < 0.05, 0.01 and 0.001, respectively. Values are means ± S.E.M.).

We next sought to determine the role of DA paracrine signalling pathways in the integrated chemosensory output by monitoring for the first time Ca^2+^ transients simultaneously in type I cells, type II cells, and petrosal afferent neurons. To this end, we attempted to reconstruct functional chemosensory circuits by coculturing type I/type II cell clusters with dissociated petrosal neurons (Figure 5B), as previously described [3,20,33]. For these studies, we only considered cases where hypercapnia-evoked Ca^2+^ transients were detectable in all three cell types during simultaneous recordings, and where there was almost full recovery of the Ca^2+^ responses after washout of sulpiride. In a few successful cases that met these criteria (i.e., three out of 18 coculture preparations), exemplified in Figure 5A, the hypercapnia-evoked Ca^2+^ transients were markedly potentiated during sulpiride application, not only in type I and type II cells as expected (see Figure 3), but also in the petrosal neuron (PN). Summary data of the hypercapnia-evoked integrated Ca^2+^ response in type I cells, type II cells, and petrosal neurons before, during, and after sulpiride are shown in Figure 5C,D,E, whereas Figure 5 H–J demonstrate the corresponding increases in duration of the response. Sulpiride caused an approximately 7×, 3×, and 4.5× increase in Ca^2+^ transients in type I cells, type II cells, and petrosal neurons respectively (Figure 5C–E; *p* < 0.05). Because isolated rat petrosal neurons cultured alone are insensitive to hypercapnia [3,34], the Ca^2+^ transients seen in cocultured petrosal neurons in Figure 5A likely arose mainly from the stimulation of postsynaptic ionotropic P2X2/3 receptors by ATP released from type I cells during hypercapnia [3,34]. However, as discussed below, type II cells may also act as a secondary source of ATP that could potentially contribute to the overall petrosal Ca^2+^ response in Figure 5A.

### 2.6. Evidence for Crosstalk between Type II Cells and Petrosal Neurons in Cocultures

As expected, and consistent with previous reports from this laboratory [3,22], the Ca^2+^ responses during hypercapnia occurred first in the type I cell, followed after a delay by responses in the petrosal neuron and type II cell (Figure 5F). Selective stimulation of P2Y2R on type II cells by UTP has previously been shown to activate carbenoxolone- and ^10^Panx peptide- sensitive pannexin-1 channels, leading to the further release of ATP which can in turn excite P2X2/3R on nearby petrosal neurons [20,35]. This raises the question whether paracrine stimulation of type II cells by ATP release from type I cells contributed to the petrosal Ca^2+^ response during hypercapnia (Figure 5A), by the mechanism of ‘ATP-induced ATP release’. Because we routinely used the P2Y2R agonist UTP to confirm the identity of type II cells, and petrosal neurons are not directly sensitive to UTP [20,35], the coculture model provided an opportunity to test whether type II cells could communicate directly with petrosal neurons. Indeed, as illustrated in Figure 5A,F, the application of UTP (100 μM) initiated Δ[Ca^2+^]_i_ responses not only in the type II cell as expected, but also in both adjacent type I cell and petrosal neuron. Crosstalk from type II to type I cells, involving adenosine generated from breakdown of extracellular ATP, likely accounted for the Ca^2+^ response in the type I cell [22]. To confirm that the UTP-evoked petrosal Ca^2+^ response in Figure 5A,F, likely arose from direct communication with type II cells, rather than an indirect route involving type I cells, we compared latencies of the type I cell and petrosal Ca^2+^ responses relative to the type II cell response. As shown in Figure 5G, the petrosal (PN) Ca^2+^ response to UTP preceded that of the type I cell response, consistent with direct communication from type II cells to the petrosal neuron.

## 3. Discussion

In this study we characterize a novel inhibitory pathway in the rat CB whereby dopamine (DA), a paracrine neuromodulator released from chemoreceptor type I cells, suppresses purinergic P2Y2R-mediated Ca^2+^ signalling in adjacent glial-like type II cells. This dopaminergic pathway is proposed to modulate the level of the excitatory neurotransmitter ATP at the CB sensory synapse by limiting the ability of type II cells to boost ATP levels via the mechanism of ‘ATP-induced ATP’ release [3,5,20]. It is now well-established that ATP is released from type I cells during chemotransduction [5,7,30,33] and, in addition to its excitatory effects at P2X2/3R on petrosal afferent terminals [33,36,37], it may cause paracrine stimulation of P2Y2R on adjacent type II cells leading to a rise in intracellular Ca^2+^ and opening of ATP-permeable pannexin-1 channels [18,19,20,35]. Based on the present study, co-released DA is proposed to activate a parallel pathway in type II cells that suppresses intracellular Ca^2+^ signalling and presumably Ca^2+^-dependent activation of pannexin-1 channels [21,35]. The inhibitory effects of DA were reversibly blocked by sulpiride, an antagonist of D2 receptors that are highly expressed in the rat CB [8,10,11], suggesting that D2 receptors (D2R) may be involved. However, sulpiride is also a D3 receptor blocker and though we cannot presently rule out a contribution from D3R, especially in light of its reported expression in goat CB [38], we are unaware of any evidence supporting D3R expression in rat CB. Recent immunohistochemical evidence supports expression of D2R on tyrosine hydroxylase-positive type I, but not S100B-positive type II, cells in the rat CB [39]. While this could argue against the involvement of D2R, firm conclusions cannot be drawn because of the potential for sampling limitations in the latter study given that DA inhibition was seen in ~75% type II cells. In addition, similar limitations would apply if there was compartmentalization in DA receptor distribution along the processes of the elongated type II cells. Based on these considerations the molecular identity of the DA-receptors mediating the inhibitory effects on type II cells remains unresolved and requires further investigation.

### 3.1. Dopaminergic Inhibition of P2Y2R-Mediated Calcium Signalling in Type II Cells

In addition to their elongated shape, type II cells were positively identified by the presence of a robust rise in intracellular Ca^2+^ when stimulated with the P2Y2R agonist UTP [18,19,20,21,27]. In the majority of those cells, co-application of DA caused a marked and reversible inhibition of the UTP-evoked intracellular Ca^2+^ rise, an effect that was prevented or opposed by sulpiride. While these studies were useful in identifying antagonistic signalling pathways that regulated Ca^2+^ transients in the same type II cell, they did not address whether endogenously-released, paracrine activators of the respective P2Y2 and DA receptors acted in a similar way. To this end, we took advantage of previous studies demonstrating crosstalk between type I and type II cells. In those studies, type I cell depolarizations triggered by chemostimuli such as hypoxia and hypercapnia, as well as high K^+^, often led to secondary, delayed Ca^2+^ elevations in neighboring type II cells [22,23]. These delayed responses were at least partly attributable to paracrine activation of P2Y2R on type II cells by ATP released from type I cells because they were inhibited by suramin (a P2Y2R antagonist) and apyrase (an ATP hydroylase) [22]. However, DA is also co-released from type I cells by the same stimuli, raising the possibility that the magnitude and frequency of the delayed Ca^2+^ responses in type II cells were suppressed by concurrent DA-mediated inhibition. This appeared to be the case since addition of sulpiride to block DA receptors, or depleting DA stores in type I cells by pre-treatment with reserpine, caused an increase in both the frequency and magnitude of the delayed type II cell responses during exposures to hypercapnia or high K^+^. The rise in intracellular Ca^2+^ following stimulation of P2Y2R is thought to involve the PLC-IP_3_-Ca^2+^ signalling pathway [40]. However, the mechanism by which DA inhibits Ca^2+^ signalling during P2YR activation in type II cells is currently unknown. In this regard, there is a report that in a subpopulation of nucleus accumbens neurons in the central nervous system, DA activation of D2R counteracts adenosine A2aR-mediated enhancement of IP_3_-dependent Ca^2+^ signalling [41]. We recently reported that another CB neuromodulator, i.e., histamine, also counteracted the P2Y2R-mediated rise in intracellular Ca^2+^ though this occurred in only a minority population (<25%) of type II cells [23].

### 3.2. Significance of DA Inhibition and Type II Cell Signalling to the Integrated Carotid Body Sensory Output

The use of CB cocultures where type I/type II cell clusters occasionally formed functional interactions with nearby petrosal neurons, combined with simultaneous Ca^2+^ imaging of all three cell types, allowed us to explore the role of DA signalling and type II cells in the integrated chemosensory response. During acute hypercapnia (10% CO_2_), type I cell Ca^2+^ elevations preceded corresponding ones in cocultured petrosal neurons and type II cells, as expected [3,22]. Notably, perfusion with sulpiride caused a marked potentiation in the magnitude of the hypercapnia-evoked Ca^2+^ responses in all three cell types. Sulpiride blockade of autocrine-paracrine D2R on type I cells likely diminished the negative feedback inhibitory influence of released DA, thereby accounting for the enhancement of the Ca^2+^ signal in those cells [11,15,16,17]. In turn, the resulting elevated Ca^2+^ signal in type I cells would be expected to contribute significantly to the enhanced petrosal Ca^2+^ response by increasing extracellular ATP and adenosine, which would stimulate postsynaptic P2X2/3R and A2aR respectively [3,5,6,7,14]. In addition, sulpiride blockade of postsynaptic D2R on petrosal afferent endings is expected to remove the inhibitory effect of released DA on cyclic nucleotide-gated, hyperpolarization-activated (HCN4) channels, resulting in increased excitability and voltage-gated Ca^2+^ entry [14].

The petrosal afferent response could also be influenced by the counteracting excitatory and inhibitory signalling pathways in type II cells. For example, ATP released from type I cells is expected to stimulate P2Y2R on neighboring type II cells, leading to a Ca^2+^-dependent activation of pannexin-1 channels and the further release of ATP [19,20,35]. However, the present data suggest that this pathway is blunted during chemoexcitation (and perhaps during basal or resting conditions) by the simultaneous release of DA which activates receptors on type II cells, leading to a reduction in the P2Y2R-mediated intracellular Ca^2+^ signal. Sulpiride blockade of those receptors led to a potentiation of the hypercapnia-evoked Ca^2+^ signal in type II cells, and presumably an increased release of ATP via pannexin-1 channels [20,35]. Therefore, the antagonistic ATP- and DA-dependent signalling pathways in type II cells may influence petrosal excitation by regulating intracellular Ca^2+^ and release of ATP. Thus, given that petrosal neurons are not directly sensitive to hypercapnia [3,34], the type II cell may actively contribute to the Ca^2+^ signal recorded in cocultured petrosal neurons during hypercapnia via release of ATP, which should be greater after sulpiride. The fate of type II cell-derived ATP is two-fold. First, it could directly activate P2X2/3R on petrosal neurons causing an increase in membrane excitability and intracellular Ca^2+^ [20]. The latter pathway is supported by the observation that in cocultures, direct activation of P2Y2R on type II cells using UTP led to a rapid petrosal Ca^2+^ signal that occurred even before neighboring type I cells responded. Second, ATP released from type II cells could be degraded by a series of ectonucleotidases in the synaptic region to adenosine [42], which could in turn directly excite A2aR on type I cells and/or petrosal endings [6,14,19,28,35,43]. Taken together, these data support the notion that the integrated sensory CB output, as reflected by petrosal chemoafferent activity, is determined by interactions involving both type I and type II cells.

### 3.3. Limitations

This study has several limitations that preclude our ability to draw firm conclusions about its overall physiological relevance in vivo. First, the experiments that led to the conclusion that DA inhibits purinergic signalling in type II cells are based on a culture model where CB cells are grown in isolation for 2–5 days. It is possible that, within this timeframe, and under the foreign in vitro conditions, phenotypic changes in type II cells may have occurred resulting in the expression of DA receptors not normally present in situ. Second, our CB cultures and co-cultures were grown in monolayers, and are therefore not an accurate representation of the in vivo three-dimensional structures, where cell-cell interactions are likely to be more favorable and more frequent. Third, it cannot be ascertained that, in our co-culture model, the dissociated petrosal neurons that formed functional connections with type I/type II cell clusters were of the identical chemosensory phenotype as their in vivo counterparts that respond to hypercapnia. This was the case because the petrosal ganglion contains a mixed neuronal population, even though care was taken during dissection to avoid contaminating nodose ganglion neurons. Fourth, our studies of synaptic and/or paracrine interactions among the three cell types in coculture were hampered by the frequent lack of an optimal spatial relationship between the petrosal soma and type I/type II cluster, as well as the added requirement that the concentration of any released neurotransmitters/neuromodulators had to reach levels sufficient to activate nearby receptors. Despite these limitations, our studies make novel predictions that await further confirmation using in vivo or ex vivo preparations. In this regard, the proposal that direct stimulation of type II cells alone, leading to a rise in intracellular Ca^2+^ and activation petrosal afferents via ATP release through pannexin-1 channels has important implications. For example, some pathophysiological conditions associated with enhanced CB drive, e.g., obesity and congestive heart failure, are also linked to elevations in circulatory levels of 5-HT and angiotensin II, which are potent stimulants of type II cells [19,21,35].

## 4. Materials and Methods

### 4.1. Ethical Approval

Animal handling and tissue removal followed the guidelines outlined by the Canadian Council on Animal Care (CCAC) and were approved by McMaster’s Animal Research Ethics Board (AREB; AUP# 16-09-33, approved 18 October 2016). Animals were held in the McMaster Central Animal Facility under constant 12:12 h light-dark cycle and had ad libitum access to food and water.

### 4.2. Carotid Body Cultures and Petrosal Neuron-Carotid Body Cocultures

Carotid body (CB) cultures and petrosal neuron-CB cocultures were prepared using procedures previously described in this laboratory [20,33,35,44]. Briefly, rat pups (9–11 days old; Wistar, Charles River, QC, Canada) were rendered unconscious with a swift blow to the back of the head and euthanized immediately via decapitation. Bifurcations of the bilateral common carotid arteries were excised prior to isolation of the CBs and removal of the surrounding connective tissue. Isolated CBs were then exposed for 1h to an enzyme solution containing 0.1% trypsin from porcine pancreas (cat # T4799; Sigma-Aldrich, Oakville, ON, Canada) and 0.1% collagenase (cat # 17018-029; Gibco, Grand Island, NY, USA). The CB tissues were then mechanically dissociated with forceps and triturated before plating on pre-coated tissue culture dishes containing a thin layer of Matrigel (BD Biosciences, Mississauga, ON, Canada). Co-cultures were produced in one of two ways: (i) dissociated petrosal ganglion neurons and dissociated CB cells (containing type I/type II cell clusters) from the same animals were plated together on the same day; or (ii) monolayers containing dissociated CB cells were first prepared and then an overlay of dissociated petrosal neurons was added 3–5 days later. The procedures for preparing dissociated petrosal neurons were similar to those described in detail elsewhere [33,44]. Cultures were maintained at 37 °C in a humidified atmosphere of 95% air-5% CO_2_. Culture media consisted of F-12 nutrient medium supplemented with 1% penicillin-streptomycin, 1% glutamine, 0.3% glucose, 3 µg/mL insulin, 5% fetal bovine serum (lot # 1048463, cat # 12483), and 5% Cosmic calf serum (Hyclone Laboratories Inc, Logan, UT, USA) as previously described [20,21]. The Ca^2+^ imaging experiments were performed on cultures that were 2–5 days old.

### 4.3. Fura-2 Measurements of Intracellular Calcium

Fura-2 measurements of intracellular Ca^2+^ concentrations ([Ca^2+^]_i_) were routinely obtained from type I and type II cells, and in some cases from cocultured petrosal neurons as well. The procedures were similar to those described in detail in previous studies from this laboratory [20,21,27]. In some experiments aimed to deplete dopamine (DA) stores, cultures were first treated with reserpine (1 μM) for ~45 min just before the start of the Ca^2+^ imaging experiments. For the latter, all cultures were loaded with 2.5 µM fura-2 AM diluted in a physiological bicarbonate-buffered saline for 30–40 min at 37 °C. Cultures were washed for 10 min to remove free dye and then imaged using a Nikon Eclipse TE2000-U inverted microscope (Nikon, Mississauga, ON, Canada) equipped with Lambda DG-4 ultra-high-speed wavelength changer (Sutter Instruments Co., Novato, CA, USA), a Hamamatsu OCRCA-ET digital CCD camera (Hamamatsu, Sewickley, PA, USA) and a Nikon S-Fluor 40× oil-immersion objective lens with a numerical aperture of 1.3. Images were acquired every 2 s at 340 nm and 380 nm excitation, with an exposure time of 100–200 ms. Pseudocolour ratiometric data were obtained using Simple PCI software version 5.3 and used to calculate the [Ca^2+^]_i_ according to the Grynkiewicz equation [22,45]. The procedures for instrument calibration and calculation of intracellular free Ca^2+^ were identical to those described previously [20,27]. As in previous studies [27], cells were considered ‘responsive’ if the intracellular Ca^2+^ transient during stimulus application was >20 nM above baseline or preceding ‘control’ intracellular Ca^2+^ level

### 4.4. Solutions and Drugs

The perfusion solution (saline) was maintained at 35–37 °C and was composed of (in mM): NaHCO_3_ (24), NaCl (115), glucose (5), KCl (5), CaCl_2_ (2) and MgCl_2_ (1) at pH ~7.4, maintained by bubbling a 5% CO_2_-95% air mixture. Isohydric hypercapnia (10% CO_2_; pH ~7.4) was generated by doubling the concentration of NaHCO_3_ to 48 mM (maintaining osmolarity by reducing NaCl to 91 mM) and aerating with 10% CO_2_–90% air (pH ∼7.4). Hypoxia was generated by bubbling standard perfusion solution (as stated above) with 5% CO_2_–90% N_2_ (pH ∼7.4). High extracellular K^+^ solution (30 mM KCl) was made by equimolar substitution of NaCl for KCl. UTP (94370), dopamine hydrochloride (H8502), (±)-sulpiride (S8010), and reserpine (R0875) were purchased from Sigma Aldrich (Saint Louis, MO, USA). Drugs were perfused over the cells for 1 min before, 1 min during, and 1 min after the stimulus was applied.

### 4.5. Statistics

For each experiment, dissociated carotid bodies (CBs) from one litter of 10–12 pups were divided and plated into four equal fractions at similar densities. For data analysis, the indicated (n) values in the text refer to the number of litters used for each experiment. Typically, 3–20 cells were sampled per dish. Statistical analysis was carried out using either one way or repeated measures ANOVA with Tukey’s post hoc tests (for non-parametric data). Student’s *t* test was used where noted. Results were considered statistically significant at *p* < 0.05.

## 5. Conclusions

**Figure 6:**
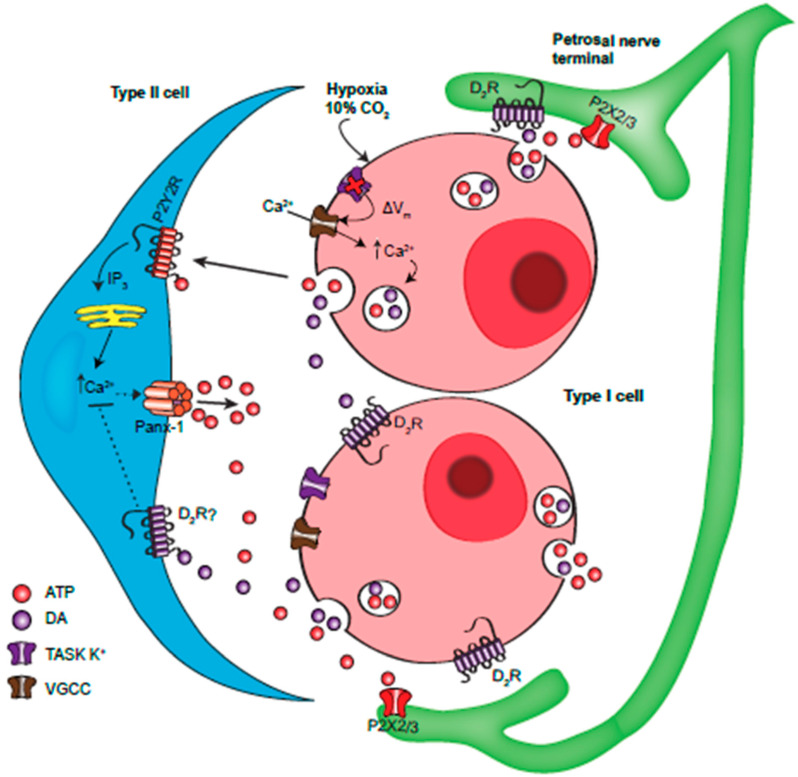
Model of the proposed interactions involving ATP and dopamine at the carotid body ‘tripartite’ synapse. During hypoxia or hypercapnia type I cells depolarize due to inhibition of Two-pore domain, Acid-Sensitive K^+^ (TASK) channels, leading to membrane depolarization, Ca^2+^ entry through voltage-gated Ca^2+^ channels (VGCC) and vesicular release of ATP and DA. ATP activates postsynaptic P2X2/3R on petrosal afferent terminals causing excitation. ATP also activates P2Y2R on adjacent type II glial cells, causing a rise in intracellular Ca^2+^ via the PLC-IP_3_-Ca^2+^ pathway. This leads to Ca^2+^-dependent opening of pannexin (Panx)-1 channels which act as conduits for the release of ATP, thereby contributing directly to petrosal excitation. Co-released DA acts on autocrine-paracrine D2 receptors on the same or adjacent type I cells, leading to a negative feedback inhibition of neurotransmitter release. DA can also act on postsynaptic D2 receptors on petrosal terminals, leading to inhibition of action potential firing. DA may also activate sulpiride-sensitive (D2 and/or D3) receptors on type II cells, leading to a blunting of the ATP-P2Y2R -mediated rise in intracellular Ca^2+^ by an unknown mechanism. In this way, DA limits the ability of type II cells to contribute to the synaptic ATP pool via the mechanism of ‘ATP-induced ATP release’ involving Panx-1 channels. Omitted for clarity are paracrine signalling pathways involving other CB neurotransmitters including adenosine, generated in part from the breakdown of extracellular ATP.

This study uncovered a previously unrecognized pathway by which dopaminergic inhibition helps control the CB chemoafferent discharge. This pathway involves the glial-like type II cells, previously proposed to contribute to CB excitation by boosting synaptic levels of the excitatory neurotransmitter ATP via the mechanism of ‘ATP-induced ATP’ release [20]. Our current model, summarized in Figure 6, purports that co-release of ATP and DA from chemoreceptor type I cells during sensory transduction results in the paracrine activation of excitatory P2Y2 and inhibitory D2-like receptors on nearby type II cells. This antagonism modulates the level of intracellular Ca^2+^ that is required for the activation of ATP-permeable pannexin-1 channels in type II cells [35]. By releasing ATP, type II cells contribute to the ‘purine pool’ that regulates excitability of petrosal chemoafferent fibers. It remains to be determined whether DA can counteract the excitatory effects of other CB modulators (e.g., angiotensin II and 5-HT) in type II cells [19,21,35].

## Abbreviations

CB	Carotid body
DA	Dopamine
D2/3R	Dopamine receptor type 2/3
Panx-1	Pannexin-1
PN	petrosal neuron
VMAT	Vesicular monoamine transporter
VGCC	Voltage-gated calcium channel
TASK	Two-pore domain, acid-sensitive K^+^
